# Transradial Prostatic Artery Embolization as a Salvage Procedure in a Patient With Severe Deformities and Refractory Hematuria of Prostatic Origin

**DOI:** 10.7759/cureus.59894

**Published:** 2024-05-08

**Authors:** Hippocrates Moschouris, Ilianna Tsetsou, Aristodimos Kaniaris, Konstantinos Stamatiou

**Affiliations:** 1 Radiology, General Hospital of Piraeus Tzaneio, Piraeus, GRC; 2 Imaging and Interventional Radiology, “Sotiria” General and Chest Diseases Hospital of Athens, Athens, GRC; 3 Urology, General Hospital of Piraeus Tzaneio, Piraeus, GRC

**Keywords:** benign prostate hyperplasia, salvage procedure, transradial access, prostatic artery embolization, refractory hematuria

## Abstract

Benign prostatic hyperplasia is a common condition causing urinary symptoms in older men. It can sometimes lead to hematuria of prostatic origin, due to increased vascularity of the enlarged gland. If this type of hematuria is severe and refractory to conservative measures, it can be life-threatening. Prostatic artery embolization (PAE) serves as a minimally invasive alternative to traditional surgical interventions, particularly in patients with comorbidities and contraindications to surgery. We present a case of a 79-year-old male with refractory hematuria of prostatic origin (RHPO), multiple comorbidities, and significant deformities of the left upper and both lower limbs. The patient was treated with PAE via the right radial artery, a less common approach in interventional radiology. The procedure was successful and led to a complete resolution of hematuria, with no complications. This report highlights the importance of adapting treatment for complex patients and shows that PAE can be safe and effective in such cases.

## Introduction

Prostatic artery embolization (PAE) is defined as selective catheterization of the prostatic arteries and injection of embolic agents, performed by interventional radiologists under angiographic guidance. It is a minimally invasive alternative to transurethral resection of the prostate (TURP) in the management of symptomatic benign prostate hyperplasia (BPH). PAE serves as a viable therapeutic option for patients exhibiting moderate to severe lower urinary tract symptoms (LUTS), who are deemed unsuitable candidates for surgical interventions primarily due to concurrent comorbidities and contraindications to anesthesia [1].

BPH is currently one of the leading causes of hematuria in males, as the hyperplastic prostatic tissue is prone to bleeding [2]. Other common causes of prostatic-origin hematuria are prostate cancer, radiation therapy, and traumatic injuries [3]. Typically, conservative measures such as catheterization are adequate for resolution. However, failure to respond to such treatments leads to refractory hematuria of prostatic origin (RHPO), which may require intensive measures, such as hospitalization and blood transfusions, and can be life-threatening [4].

Since the 1970s, PAE has been employed as a salvage therapy for patients with RHPO [5]. Unlike other visceral organs, prostatic hematuria typically lacks a distinct bleeding source, prompting PAE to target the entire gland through (ideally) bilateral embolization of the prostatic arteries [4]. Usually, PAE is performed through a transfemoral approach. Transradial access (TRA) is another alternative, mostly utilized when transfemoral access is unavailable. It is associated with fewer access-site complications; it is more comfortable for the patients and reduces total hospitalization time and overall cost [6]. We present a case where transradial PAE was deemed the only treatment option in an elderly male with RHPO, multiple comorbidities, and severe body deformities that ruled out both transfemoral PAE and standard urologic surgical management.

## Case presentation

**Figure 1:**
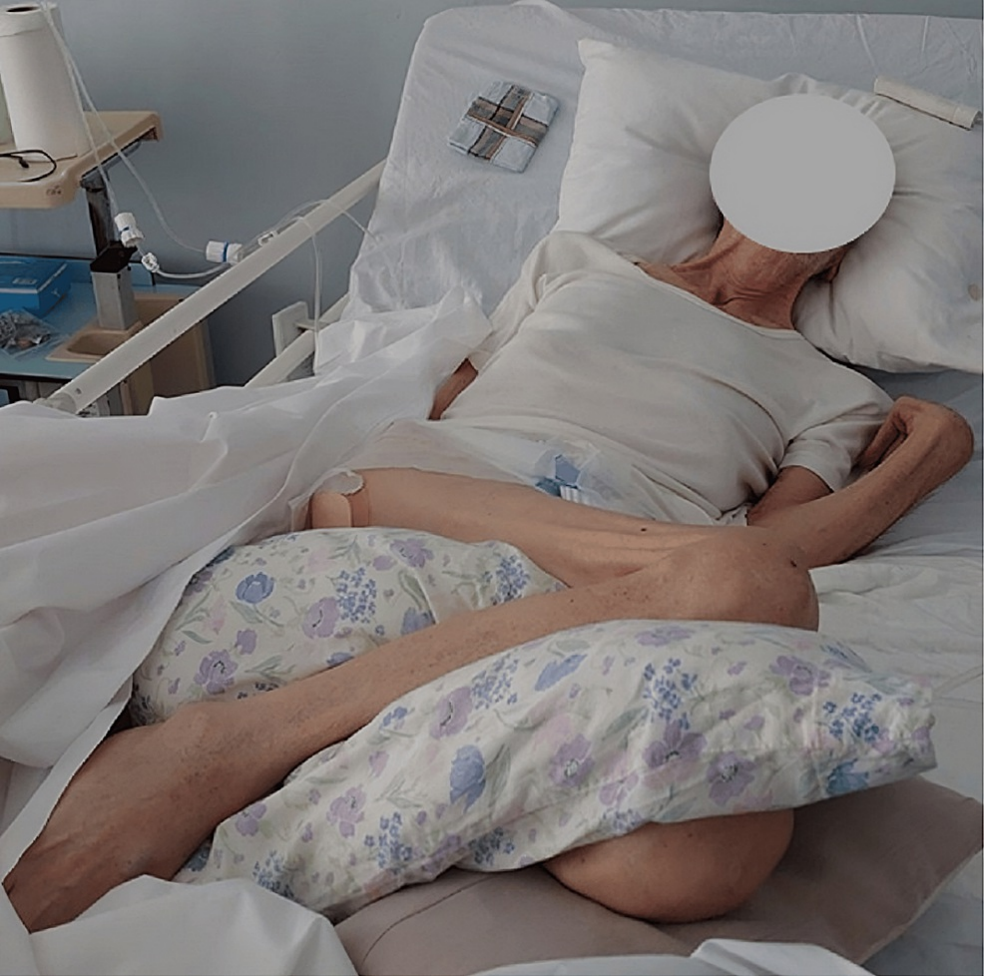
Clinical image Clinical image showing the deformity and spasticity of both lower limbs and the left upper limb of the patient, precluding femoral and left radial arterial access

A 79-year-old male presented to the emergency department with gross hematuria for a week. He had a history of heart failure, BPH, and a permanent bladder catheter due to recurrent urinary tract obstructions. Furthermore, he was chronically bedbound after suffering multiple ischemic strokes, resulting in marked spasticity affecting his left upper and both lower limbs, body deformities (Figure 1), and pressure ulcers (Karnofsky performance status score: 40).

Upon clinical assessment, tachycardia was noted, while other vital signs were within normal limits. Laboratory investigations revealed normocytic anemia [hemoglobin: 8 g/dl, hematocrit: 26%, mean corpuscular volume (MCV): 82 fL] and hyponatremia (127 meq/l), with all other parameters falling within the normal range. Transabdominal ultrasonography revealed significant prostatic enlargement (prostate volume: 125 ml). He was admitted to the hospital and initially managed conservatively with blood transfusions and saline infusions. Despite these measures, his persistent gross hematuria necessitated a second blood transfusion after two days. Given the patient’s severe deformities and frailty, he was deemed unsuitable for surgery (both transabdominal and transurethral); the patient was subsequently referred to the interventional radiology service for salvage PAE.

The patient was transferred to the angiography suite (Axiom-Artis Zee, Siemens Healthineers, Erlangen, Germany) and was administered prophylactic antibiotics (cefoxitin 2 g), analgesia (paracetamol 1 g), and gastroprotective agents (omeprazole 20 mg). Due to bilateral lower and left upper limb spasticity, femoral and left radial access was unavailable, and hence the procedure was performed through the right radial artery. Following local anesthesia, the right radial artery was catheterized under ultrasonographic guidance using a 21 G needle and 5 Fr radial sheath (Prelude, Merit Medical, Bengaluru, India). A mixture of 1000 u of unfractionated heparin and 0.2 mg nitroglycerine was administered through the sheath to prevent vasospasm.

**Figure 2:**
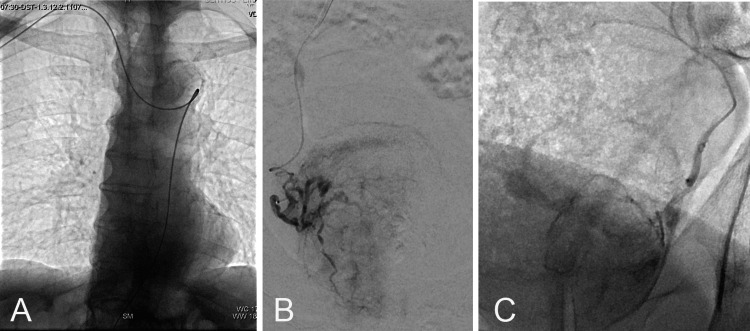
Representative angiographic images from the intervention A: Fluoroscopic image showing the passage of the guidewire through the right subclavian, right innominate artery, and thoracic aorta. B, C: Angiographic images after superselective catheterization of the prostatic arteries with microcatheter, showing a blush of the right and the left prostatic lobe, respectively

After passing through several tortuosities of the anonymous artery, the aorta (Figure 2A), and the common iliac arteries, the internal iliac arteries (IIAs) were catheterized using a 125 cm, 5 Fr “multipurpose” angiographic catheter (Merit Medical) and a curved-tip hydrophilic guidewire (Glidewire, Terumo Corp, Tokyo, Japan). Digital subtraction angiography (DSA) of each IIA was performed from this position with a manual injection of iodine contrast (20 ml) on frontal and ipsilateral oblique projections, revealing a right PA origin from the internal pudendal artery and a left PA origin from a gluteopudendal trunk. Selective catheterization of the PAs was achieved using a microcatheter (1.98 Fr, Parkway Soft, Asahi Intecc, Tokyo, Japan) and microguidewire (0.016’’ Asahi Meister Double Angle, Asahi Intecc), followed by a repeat DSA to confirm prostatic blush and absence of dangerous anastomoses. (Figures 2B, 2C). Embolization was then performed, utilizing highly compressible microspheres (Embosoft, Scitech Medical, Aparecida de Goiânia, Brazil; diameter: 100-300 μm). One and a half vial (3 ml in total) of embolic agent was required for complete flow stasis in both PAs.

Following the procedure, hemostasis was achieved using a radial compression device (PreludeSYNC, Merit Medical), and the patient was observed in the ward. Analgesics, antibiotics, and intravenous fluids were administered as part of post-procedural care. Complete resolution of hematuria was achieved the next day. The patient remained hemodynamically stable throughout hospitalization, without requiring further transfusions. No complications were observed and he was discharged home with the permanent bladder catheter in place. No recurrence of hematuria was noticed until the patient’s death after a heart attack three months post-PAE.

## Discussion

PAE has been established as a valid and effective alternative to TURP for treating symptomatic BPH; several meta-analyses have demonstrated its safety and clinical success [7-9]. It has been included in the National Institute of Health and Care Excellence (NICE) guidelines since 2018 [10] and the European Association of Urology (EAU) guidelines since 2021 [11], and was incorporated into the American Urological Association (AUA) guidelines in 2023 [12]. Its primary advantage lies in its safety, which is particularly observed in patients with multiple comorbidities, contraindications to surgical procedures, bleeding risk, and frailty [13]. To date, most PAE complications have been reportedly minor and include urinary tract infections, urinary retention, dysuria, hematuria, and hematospermia, while major complications, such as non-target embolization with subsequent organ ischemia are rare [1]. TURP is also reported to carry a small risk of complications such as blood loss requiring transfusion, clot retention, bladder neck stenosis, and urethral stricture, whereas there are no such reports associated with PAE [2].

RHPO can be managed either conservatively, surgically, or via PAE. Conservative measures include continuous bladder irrigation and bladder instillation of various agents such as silver nitrate, while surgical treatments can be either endoscopic or open [3]. In our case, due to multiple comorbidities, deformities, and general frailty, the patient was deemed unsuitable for any type of urologic surgery; therefore, PAE was offered as a life-saving treatment option. Its established role as salvage therapy for RHPO is well documented [14].

While the radial artery has been extensively used in interventional cardiology, its adoption in interventional radiology remains less common. Its main advantages over the femoral artery include reduced access site-associated bleeding complications, expedited ambulation, shorter hospitalization duration, and cost-effectiveness [15]. Its superficial location and ease of access facilitate early detection of local complications and aid in its compression [6]. While typically the left radial artery is preferred for interventions below the diaphragm to avoid maneuvers near the origins of the arch vessels [16], in our case, the right radial artery was the only available access site due to the permanent flexion of the joints of both the lower and the left upper limb. Another option would be the brachial artery; however, compared to radial, brachial artery access is associated with increased bleeding complications. The latter are also more likely to be clinically significant and require intervention [17].

Many embolic agents have been used in PAE, such as polyvinyl alcohol particles, tris-acryl gelatin microspheres, and hydrogel microspheres with coating. Embosoft, a newer, highly compressible spherical embolic agent, has not been extensively utilized in PAE. In our case, we attained the most favorable therapeutic outcome using only microspheres with a diameter of 100-300 μm.

## Conclusions

We presented a case of a frail patient with RHPO, with severe deformities and spasticity, who was unable to undergo any surgical treatment or PAE through the femoral or left radial artery. PAE with right radial access was employed to successfully treat hematuria, without any complications. This case highlights not only the safety and efficacy of PAE in fragile patients but also the versatility of this modality and the importance of tailored vascular access strategies to optimize procedural outcomes.
